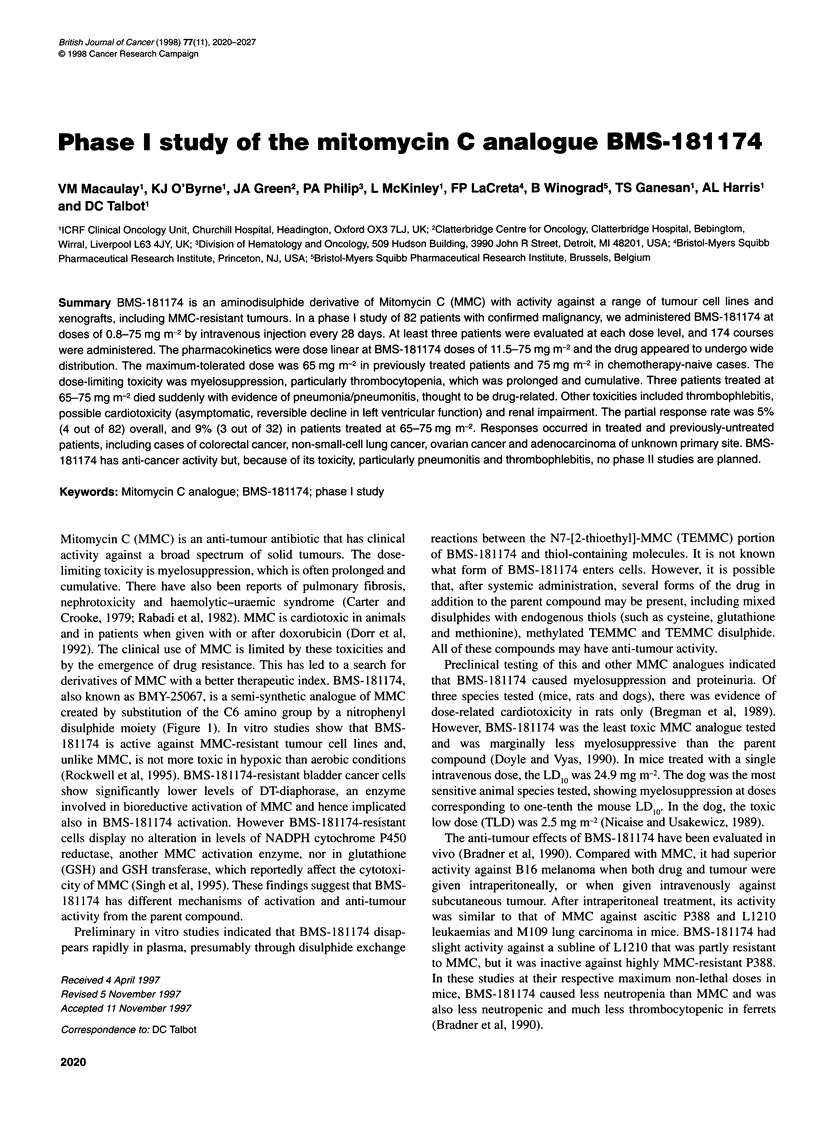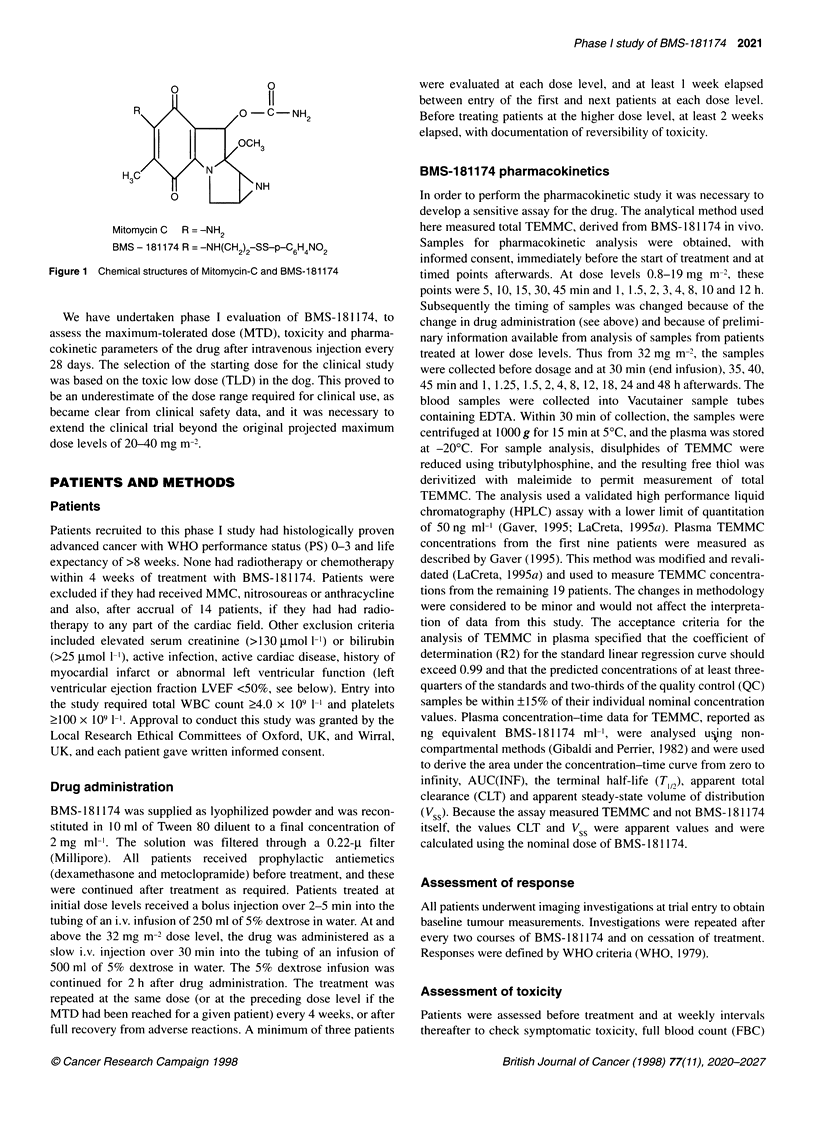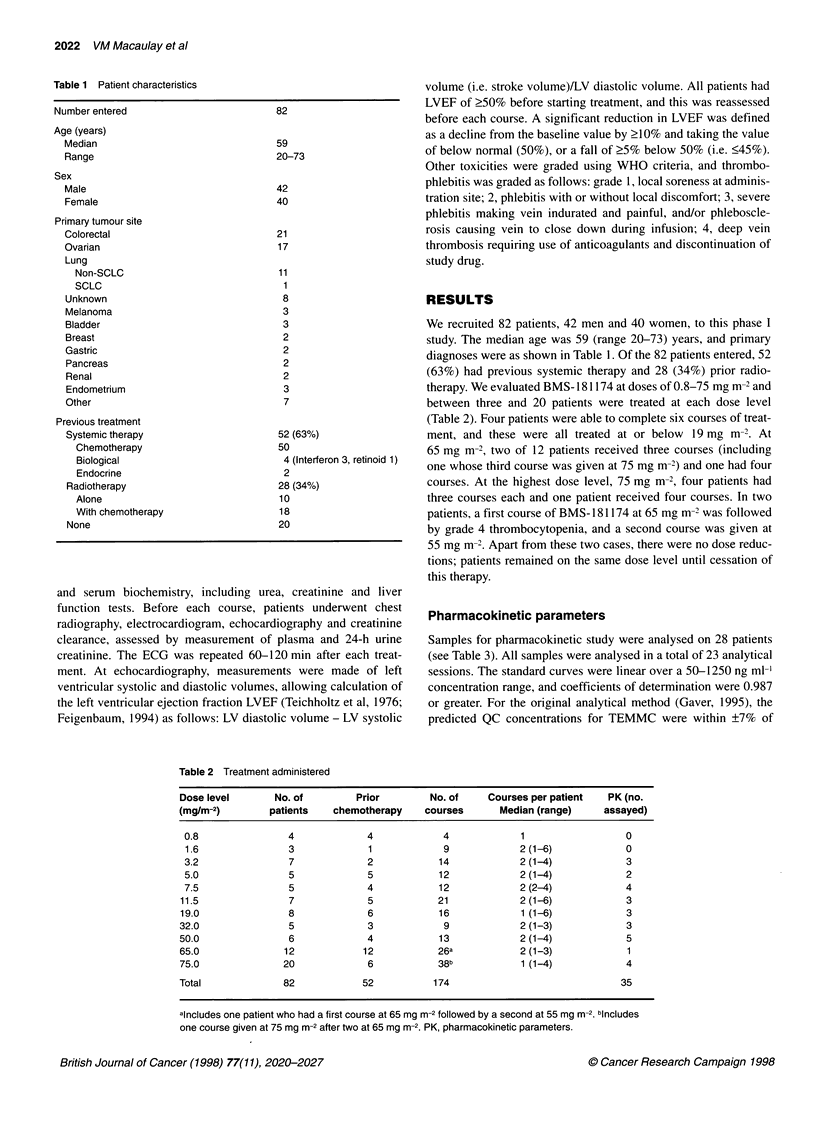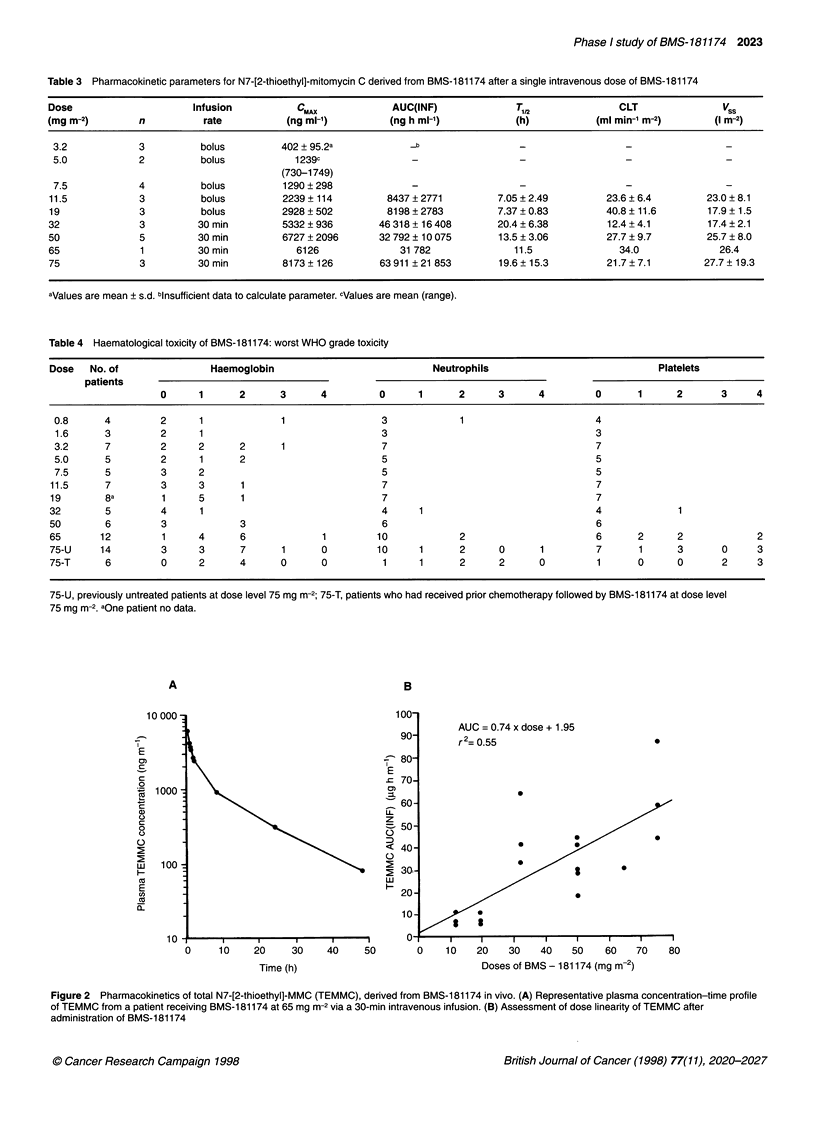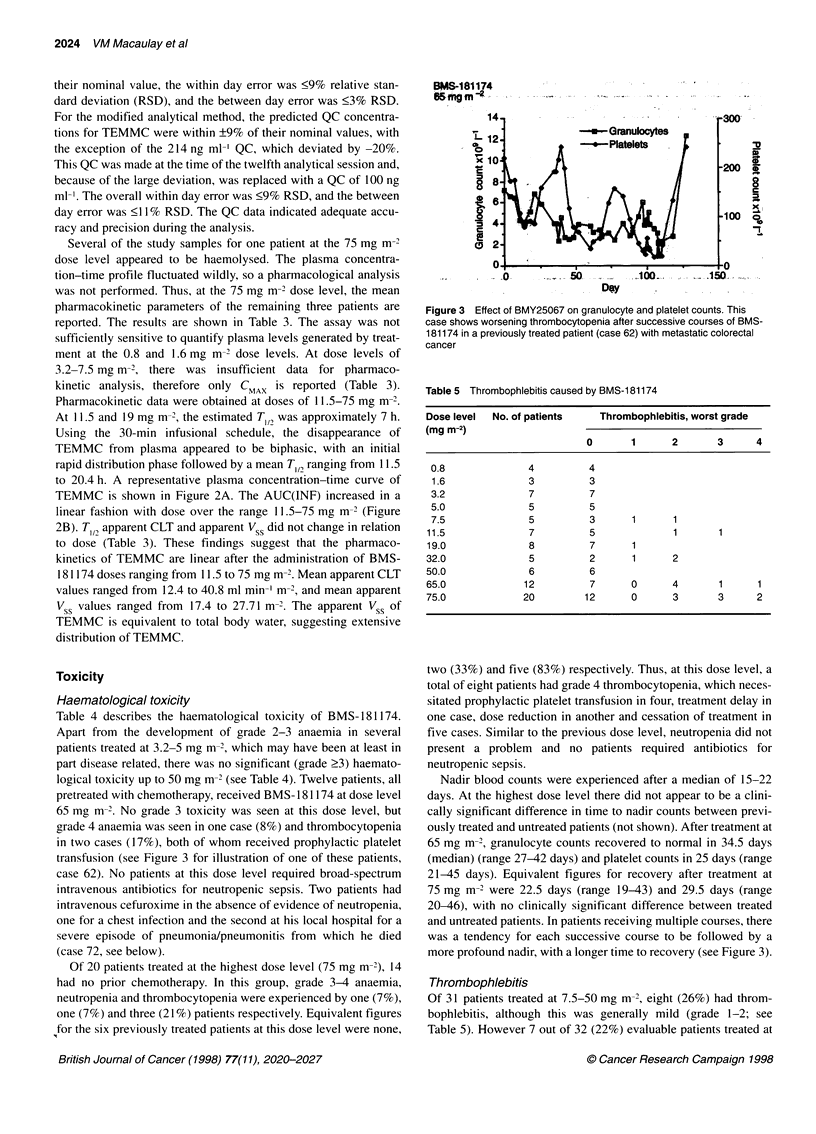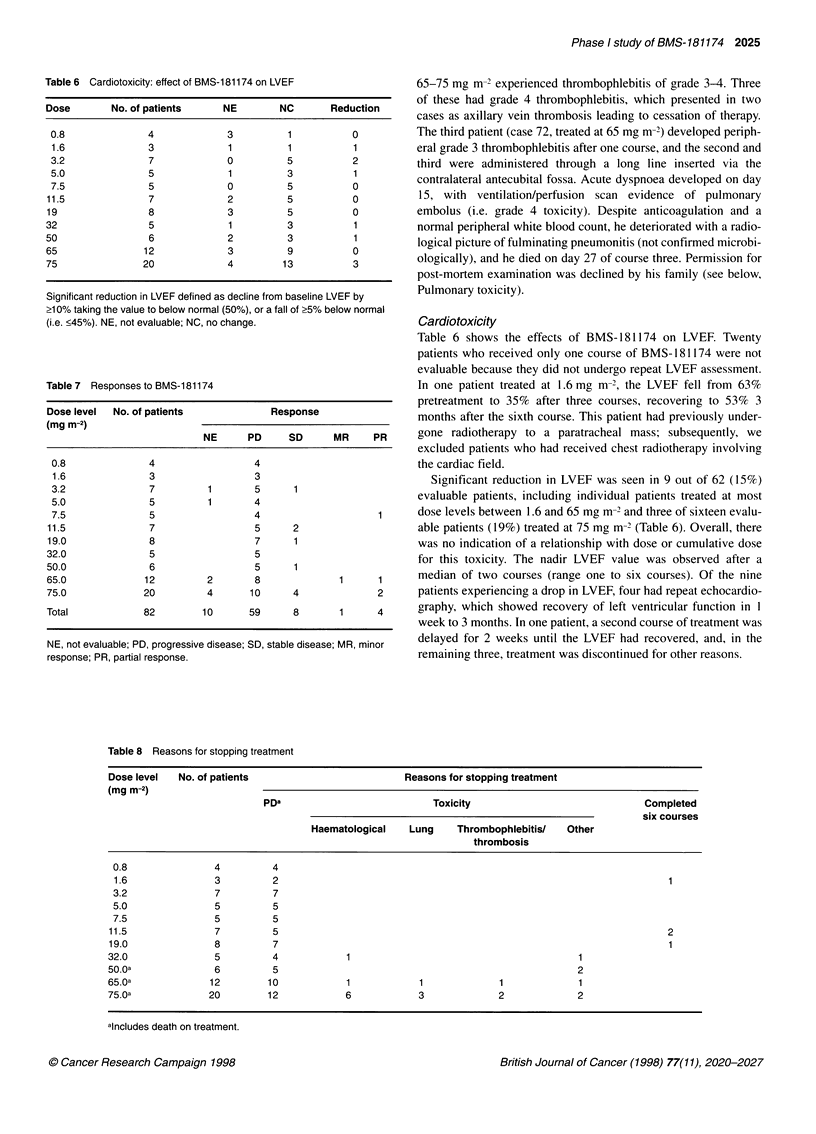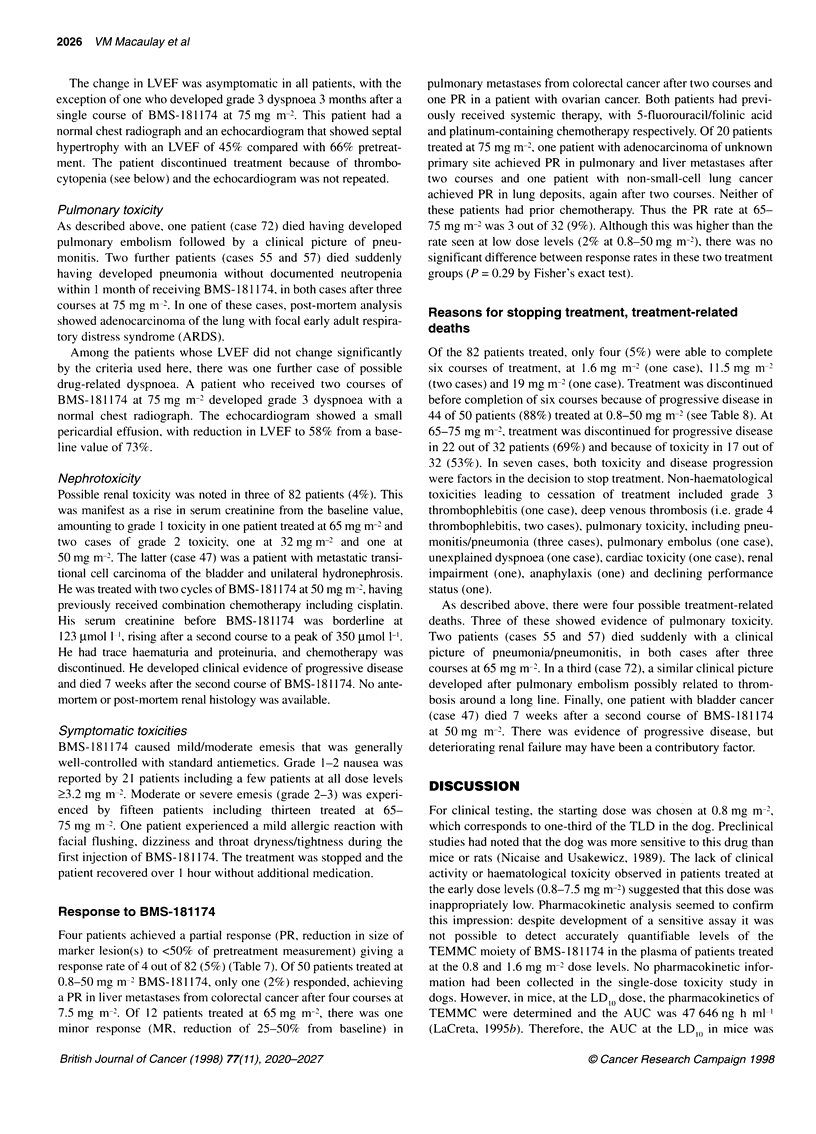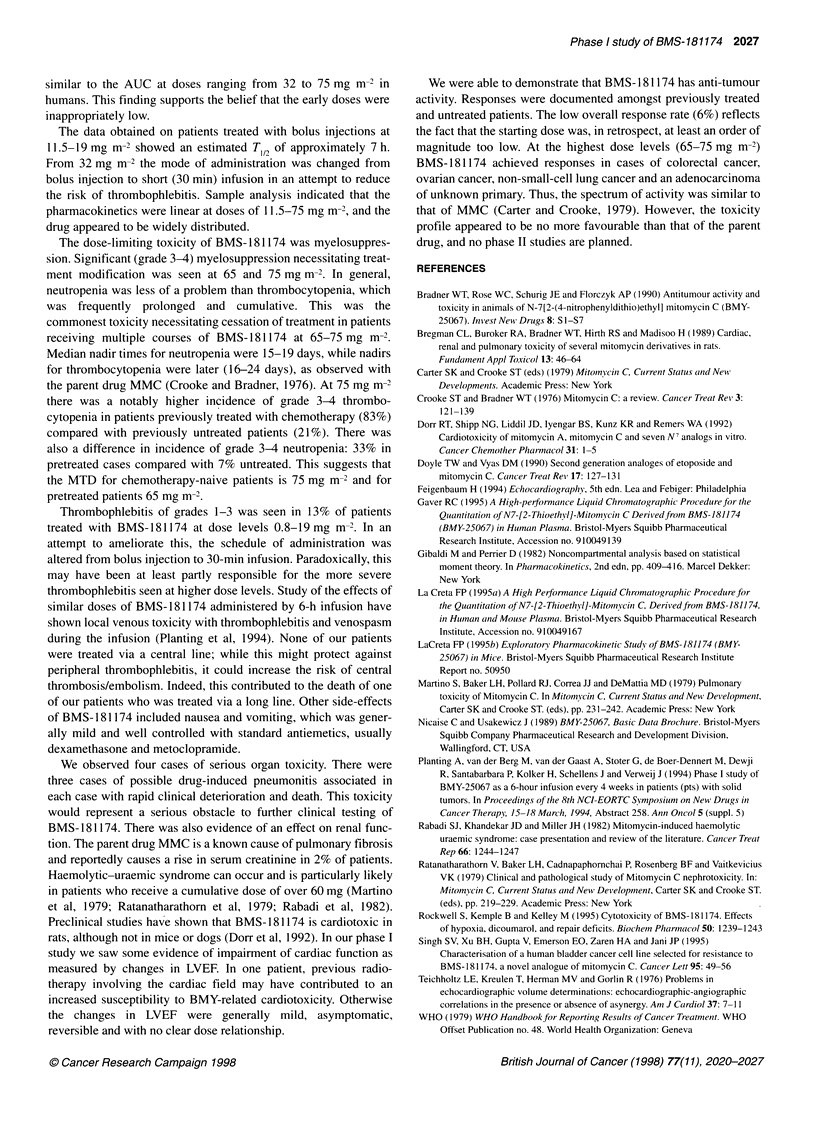# Phase I study of the mitomycin C analogue BMS-181174.

**DOI:** 10.1038/bjc.1998.336

**Published:** 1998-06

**Authors:** V. M. Macaulay, K. J. O'Byrne, J. A. Green, P. A. Philip, L. McKinley, F. P. LaCreta, B. Winograd, T. S. Ganesan, A. L. Harris, D. C. Talbot

**Affiliations:** ICRF Clinical Oncology Unit, Churchill Hospital, Headington, Oxford, UK.

## Abstract

BMS-181174 is an aminodisulphide derivative of Mitomycin C (MMC) with activity against a range of tumour cell lines and xenografts, including MMC-resistant tumours. In a phase I study of 82 patients with confirmed malignancy, we administered BMS-181174 at doses of 0.8-75 mg m(-2) by intravenous injection every 28 days. At least three patients were evaluated at each dose level, and 174 courses were administered. The pharmacokinetics were dose linear at BMS-181174 doses of 11.5-75 mg m(-2) and the drug appeared to undergo wide distribution. The maximum-tolerated dose was 65 mg m(-2) in previously treated patients and 75 mg m(-2) in chemotherapy-naive cases. The dose-limiting toxicity was myelosuppression, particularly thrombocytopenia, which was prolonged and cumulative. Three patients treated at 65-75 mg m(-2) died suddenly with evidence of pneumonia/pneumonitis, thought to be drug-related. Other toxicities included thrombophlebitis, possible cardiotoxicity (asymptomatic, reversible decline in left ventricular function) and renal impairment. The partial response rate was 5% (4 out of 82) overall, and 9% (3 out of 32) in patients treated at 65-75 mg m(-2). Responses occurred in treated and previously-untreated patients, including cases of colorectal cancer, non-small-cell lung cancer, ovarian cancer and adenocarcinoma of unknown primary site. BMS-181174 has anti-cancer activity but, because of its toxicity, particularly pneumonitis and thrombophlebitis, no phase II studies are planned.


British Journal of Cancer (1998) 77(11), 2020–2027
© 1998 Cancer Research Campaign

### Phase I study of the mitomycin C analogue BMS-181174

**VM Macaulay[1], KJ O'Byrne[1], JA Green[2], PA Philip[3], L McKinley[1], FP LaCreta[4], B Winograd[5], TS Ganesan[1], AL Harris[1] and DC Talbot[1]**

[1]ICRF Clinical Oncology Unit, Churchill Hospital, Headington, Oxford OX3 7LJ, UK; [2]Clatterbridge Centre for Oncology, Clatterbridge Hospital, Bebingtom, Wirral, Liverpool L63 4JY, UK; [3]Division of Hematology and Oncology, 509 Hudson Building, 3990 John R Street, Detroit, MI 48201, USA; [4]Bristol-Myers Squibb Pharmaceutical Research Institute, Princeton, NJ, USA; [5]Bristol-Myers Squibb Pharmaceutical Research Institute, Brussels, Belgium

**Summary** BMS-181174 is an aminodisulphide derivative of Mitomycin C (MMC) with activity against a range of tumour cell lines and xenografts, including MMC-resistant tumours. In a phase I study of 82 patients with confirmed malignancy, we administered BMS-181174 at doses of 0.8–75 mg $m^{-2}$ by intravenous injection every 28 days. At least three patients were evaluated at each dose level, and 174 courses were administered. The pharmacokinetics were dose linear at BMS-181174 doses of 11.5–75 mg $m^{-2}$ and the drug appeared to undergo wide distribution. The maximum-tolerated dose was 65 mg $m^{-2}$ in previously treated patients and 75 mg $m^{-2}$ in chemotherapy-naive cases. The dose-limiting toxicity was myelosuppression, particularly thrombocytopenia, which was prolonged and cumulative. Three patients treated at 65–75 mg $m^{-2}$ died suddenly with evidence of pneumonia/pneumonitis, thought to be drug-related. Other toxicities included thrombophlebitis, possible cardiotoxicity (asymptomatic, reversible decline in left ventricular function) and renal impairment. The partial response rate was 5% (4 out of 82) overall, and 9% (3 out of 32) in patients treated at 65–75 mg $m^{-2}$. Responses occurred in treated and previously-untreated patients, including cases of colorectal cancer, non-small-cell lung cancer, ovarian cancer and adenocarcinoma of unknown primary site. BMS-181174 has anti-cancer activity but, because of its toxicity, particularly pneumonitis and thrombophlebitis, no phase II studies are planned.

**Keywords:** Mitomycin C analogue; BMS-181174; phase I study

Mitomycin C (MMC) is an anti-tumour antibiotic that has clinical activity against a broad spectrum of solid tumours. The dose-limiting toxicity is myelosuppression, which is often prolonged and cumulative. There have also been reports of pulmonary fibrosis, nephrotoxicity and haemolytic–uraemic syndrome (Carter and Crooke, 1979; Rabadi et al, 1982). MMC is cardiotoxic in animals and in patients when given with or after doxorubicin (Dorr et al, 1992). The clinical use of MMC is limited by these toxicities and by the emergence of drug resistance. This has led to a search for derivatives of MMC with a better therapeutic index. BMS-181174, also known as BMY-25067, is a semi-synthetic analogue of MMC created by substitution of the C6 amino group by a nitrophenyl disulphide moiety (Figure 1). In vitro studies show that BMS-181174 is active against MMC-resistant tumour cell lines and, unlike MMC, is not more toxic in hypoxic than aerobic conditions (Rockwell et al, 1995). BMS-181174-resistant bladder cancer cells show significantly lower levels of DT-diaphorase, an enzyme involved in bioreductive activation of MMC and hence implicated also in BMS-181174 activation. However BMS-181174-resistant cells display no alteration in levels of NADPH cytochrome P450 reductase, another MMC activation enzyme, nor in glutathione (GSH) and GSH transferase, which reportedly affect the cytotoxicity of MMC (Singh et al, 1995). These findings suggest that BMS-181174 has different mechanisms of activation and anti-tumour activity from the parent compound.

Preliminary in vitro studies indicated that BMS-181174 disappears rapidly in plasma, presumably through disulphide exchange reactions between the N7-[2-thioethyl]-MMC (TEMMC) portion of BMS-181174 and thiol-containing molecules. It is not known what form of BMS-181174 enters cells. However, it is possible that, after systemic administration, several forms of the drug in addition to the parent compound may be present, including mixed disulphides with endogenous thiols (such as cysteine, glutathione and methionine), methylated TEMMC and TEMMC disulphide. All of these compounds may have anti-tumour activity.

Preclinical testing of this and other MMC analogues indicated that BMS-181174 caused myelosuppression and proteinuria. Of three species tested (mice, rats and dogs), there was evidence of dose-related cardiotoxicity in rats only (Bregman et al, 1989). However, BMS-181174 was the least toxic MMC analogue tested and was marginally less myelosuppressive than the parent compound (Doyle and Vyas, 1990). In mice treated with a single intravenous dose, the $LD_{10}$ was 24.9 mg $m^{-2}$. The dog was the most sensitive animal species tested, showing myelosuppression at doses corresponding to one-tenth the mouse $LD_{10}$. In the dog, the toxic low dose (TLD) was 2.5 mg $m^{-2}$ (Nicaise and Usakewicz, 1989).

The anti-tumour effects of BMS-181174 have been evaluated in vivo (Bradner et al, 1990). Compared with MMC, it had superior activity against B16 melanoma when both drug and tumour were given intraperitoneally, or when given intravenously against subcutaneous tumour. After intraperitoneal treatment, its activity was similar to that of MMC against ascitic P388 and L1210 leukaemias and M109 lung carcinoma in mice. BMS-181174 had slight activity against a subline of L1210 that was partly resistant to MMC, but it was inactive against highly MMC-resistant P388. In these studies at their respective maximum non-lethal doses in mice, BMS-181174 caused less neutropenia than MMC and was also less neutropenic and much less thrombocytopenic in ferrets (Bradner et al, 1990).

*Received 4 April 1997*
*Revised 5 November 1997*
*Accepted 11 November 1997*

*Correspondence to:* DC Talbot

Mitomycin C  R = $-NH_2$

BMS – 181174 R = $-NH(CH_2)_2-SS-p-C_6H_4NO_2$

**Figure 1** Chemical structures of Mitomycin-C and BMS-181174

We have undertaken phase I evaluation of BMS-181174, to assess the maximum-tolerated dose (MTD), toxicity and pharmacokinetic parameters of the drug after intravenous injection every 28 days. The selection of the starting dose for the clinical study was based on the toxic low dose (TLD) in the dog. This proved to be an underestimate of the dose range required for clinical use, as became clear from clinical safety data, and it was necessary to extend the clinical trial beyond the original projected maximum dose levels of 20–40 mg $m^{-2}$.

### PATIENTS AND METHODS

### Patients

Patients recruited to this phase I study had histologically proven advanced cancer with WHO performance status (PS) 0–3 and life expectancy of >8 weeks. None had radiotherapy or chemotherapy within 4 weeks of treatment with BMS-181174. Patients were excluded if they had received MMC, nitrosoureas or anthracycline and also, after accrual of 14 patients, if they had had radiotherapy to any part of the cardiac field. Other exclusion criteria included elevated serum creatinine (>130 μmol $l^{-1}$) or bilirubin (>25 μmol $l^{-1}$), active infection, active cardiac disease, history of myocardial infarct or abnormal left ventricular function (left ventricular ejection fraction LVEF <50%, see below). Entry into the study required total WBC count ≥4.0 × $10^9$ $l^{-1}$ and platelets ≥100 × $10^9$ $l^{-1}$. Approval to conduct this study was granted by the Local Research Ethical Committees of Oxford, UK, and Wirral, UK, and each patient gave written informed consent.

### Drug administration

BMS-181174 was supplied as lyophilized powder and was reconstituted in 10 ml of Tween 80 diluent to a final concentration of 2 mg $ml^{-1}$. The solution was filtered through a 0.22-μ filter (Millipore). All patients received prophylactic antiemetics (dexamethasone and metoclopramide) before treatment, and these were continued after treatment as required. Patients treated at initial dose levels received a bolus injection over 2–5 min into the tubing of an i.v. infusion of 250 ml of 5% dextrose in water. At and above the 32 mg $m^{-2}$ dose level, the drug was administered as a slow i.v. injection over 30 min into the tubing of an infusion of 500 ml of 5% dextrose in water. The 5% dextrose infusion was continued for 2 h after drug administration. The treatment was repeated at the same dose (or at the preceding dose level if the MTD had been reached for a given patient) every 4 weeks, or after full recovery from adverse reactions. A minimum of three patients were evaluated at each dose level, and at least 1 week elapsed between entry of the first and next patients at each dose level. Before treating patients at the higher dose level, at least 2 weeks elapsed, with documentation of reversibility of toxicity.

### BMS-181174 pharmacokinetics

In order to perform the pharmacokinetic study it was necessary to develop a sensitive assay for the drug. The analytical method used here measured total TEMMC, derived from BMS-181174 in vivo. Samples for pharmacokinetic analysis were obtained, with informed consent, immediately before the start of treatment and at timed points afterwards. At dose levels 0.8–19 mg $m^{-2}$, these points were 5, 10, 15, 30, 45 min and 1, 1.5, 2, 3, 4, 8, 10 and 12 h. Subsequently the timing of samples was changed because of the change in drug administration (see above) and because of preliminary information available from analysis of samples from patients treated at lower dose levels. Thus from 32 mg $m^{-2}$, the samples were collected before dosage and at 30 min (end infusion), 35, 40, 45 min and 1, 1.25, 1.5, 2, 4, 8, 12, 18, 24 and 48 h afterwards. The blood samples were collected into Vacutainer sample tubes containing EDTA. Within 30 min of collection, the samples were centrifuged at 1000 ***g*** for 15 min at 5°C, and the plasma was stored at –20°C. For sample analysis, disulphides of TEMMC were reduced using tributylphosphine, and the resulting free thiol was derivitized with maleimide to permit measurement of total TEMMC. The analysis used a validated high performance liquid chromatography (HPLC) assay with a lower limit of quantitation of 50 ng $ml^{-1}$ (Gaver, 1995; LaCreta, 1995*a*). Plasma TEMMC concentrations from the first nine patients were measured as described by Gaver (1995). This method was modified and revalidated (LaCreta, 1995*a*) and used to measure TEMMC concentrations from the remaining 19 patients. The changes in methodology were considered to be minor and would not affect the interpretation of data from this study. The acceptance criteria for the analysis of TEMMC in plasma specified that the coefficient of determination (R2) for the standard linear regression curve should exceed 0.99 and that the predicted concentrations of at least three-quarters of the standards and two-thirds of the quality control (QC) samples be within ±15% of their individual nominal concentration values. Plasma concentration–time data for TEMMC, reported as ng equivalent BMS-181174 $ml^{-1}$, were analysed using non-compartmental methods (Gibaldi and Perrier, 1982) and were used to derive the area under the concentration–time curve from zero to infinity, AUC(INF), the terminal half-life ($T_{1/2}$), apparent total clearance (CLT) and apparent steady-state volume of distribution ($V_{SS}$). Because the assay measured TEMMC and not BMS-181174 itself, the values CLT and $V_{SS}$ were apparent values and were calculated using the nominal dose of BMS-181174.

### Assessment of response

All patients underwent imaging investigations at trial entry to obtain baseline tumour measurements. Investigations were repeated after every two courses of BMS-181174 and on cessation of treatment. Responses were defined by WHO criteria (WHO, 1979).

### Assessment of toxicity

Patients were assessed before treatment and at weekly intervals thereafter to check symptomatic toxicity, full blood count (FBC)

© Cancer Research Campaign 1998

**Table 1** Patient characteristics

| | |
|---|---|
| Number entered | 82 |
| Age (years) | |
| Median | 59 |
| Range | 20–73 |
| Sex | |
| Male | 42 |
| Female | 40 |
| Primary tumour site | |
| Colorectal | 21 |
| Ovarian | 17 |
| Lung | |
| Non-SCLC | 11 |
| SCLC | 1 |
| Unknown | 8 |
| Melanoma | 3 |
| Bladder | 3 |
| Breast | 2 |
| Gastric | 2 |
| Pancreas | 2 |
| Renal | 2 |
| Endometrium | 3 |
| Other | 7 |
| Previous treatment | |
| Systemic therapy | 52 (63%) |
| Chemotherapy | 50 |
| Biological | 4 (Interferon 3, retinoid 1) |
| Endocrine | 2 |
| Radiotherapy | 28 (34%) |
| Alone | 10 |
| With chemotherapy | 18 |
| None | 20 |

and serum biochemistry, including urea, creatinine and liver function tests. Before each course, patients underwent chest radiography, electrocardiogram, echocardiography and creatinine clearance, assessed by measurement of plasma and 24-h urine creatinine. The ECG was repeated 60–120 min after each treatment. At echocardiography, measurements were made of left ventricular systolic and diastolic volumes, allowing calculation of the left ventricular ejection fraction LVEF (Teichholtz et al, 1976; Feigenbaum, 1994) as follows: LV diastolic volume – LV systolic volume (i.e. stroke volume)/LV diastolic volume. All patients had LVEF of ≥50% before starting treatment, and this was reassessed before each course. A significant reduction in LVEF was defined as a decline from the baseline value by ≥10% and taking the value of below normal (50%), or a fall of ≥5% below 50% (i.e. ≤45%). Other toxicities were graded using WHO criteria, and thrombophlebitis was graded as follows: grade 1, local soreness at administration site; 2, phlebitis with or without local discomfort; 3, severe phlebitis making vein indurated and painful, and/or phlebosclerosis causing vein to close down during infusion; 4, deep vein thrombosis requiring use of anticoagulants and discontinuation of study drug.

### RESULTS

We recruited 82 patients, 42 men and 40 women, to this phase I study. The median age was 59 (range 20–73) years, and primary diagnoses were as shown in Table 1. Of the 82 patients entered, 52 (63%) had previous systemic therapy and 28 (34%) prior radiotherapy. We evaluated BMS-181174 at doses of 0.8–75 mg $m^{-2}$ and between three and 20 patients were treated at each dose level (Table 2). Four patients were able to complete six courses of treatment, and these were all treated at or below 19 mg $m^{-2}$. At 65 mg $m^{-2}$, two of 12 patients received three courses (including one whose third course was given at 75 mg $m^{-2}$) and one had four courses. At the highest dose level, 75 mg $m^{-2}$, four patients had three courses each and one patient received four courses. In two patients, a first course of BMS-181174 at 65 mg $m^{-2}$ was followed by grade 4 thrombocytopenia, and a second course was given at 55 mg $m^{-2}$. Apart from these two cases, there were no dose reductions; patients remained on the same dose level until cessation of this therapy.

### Pharmacokinetic parameters

Samples for pharmacokinetic study were analysed on 28 patients (see Table 3). All samples were analysed in a total of 23 analytical sessions. The standard curves were linear over a 50–1250 ng $ml^{-1}$ concentration range, and coefficients of determination were 0.987 or greater. For the original analytical method (Gaver, 1995), the predicted QC concentrations for TEMMC were within ±7% of

**Table 2** Treatment administered

| Dose level (mg/$m^{-2}$) | No. of patients | Prior chemotherapy | No. of courses | Courses per patient Median (range) | PK (no. assayed) |
|---|---|---|---|---|---|
| 0.8 | 4 | 4 | 4 | 1 | 0 |
| 1.6 | 3 | 1 | 9 | 2 (1–6) | 0 |
| 3.2 | 7 | 2 | 14 | 2 (1–4) | 3 |
| 5.0 | 5 | 5 | 12 | 2 (1–4) | 2 |
| 7.5 | 5 | 4 | 12 | 2 (2–4) | 4 |
| 11.5 | 7 | 5 | 21 | 2 (1–6) | 3 |
| 19.0 | 8 | 6 | 16 | 1 (1–6) | 3 |
| 32.0 | 5 | 3 | 9 | 2 (1–3) | 3 |
| 50.0 | 6 | 4 | 13 | 2 (1–4) | 5 |
| 65.0 | 12 | 12 | 26[a] | 2 (1–3) | 1 |
| 75.0 | 20 | 6 | 38[b] | 1 (1–4) | 4 |
| Total | 82 | 52 | 174 | | 35 |

[a]Includes one patient who had a first course at 65 mg $m^{-2}$ followed by a second at 55 mg $m^{-2}$. [b]Includes one course given at 75 mg $m^{-2}$ after two at 65 mg $m^{-2}$. PK, pharmacokinetic parameters.

© Cancer Research Campaign 1998

**Table 3** Pharmacokinetic parameters for N7-[2-thioethyl]-mitomycin C derived from BMS-181174 after a single intravenous dose of BMS-181174

| Dose (mg m⁻²) | $n$ | Infusion rate | $C_{MAX}$ (ng ml⁻¹) | AUC(INF) (ng h ml⁻¹) | $T_{1/2}$ (h) | CLT (ml min⁻¹ m⁻²) | $V_{ss}$ (l m⁻²) |
|---|---|---|---|---|---|---|---|
| 3.2 | 3 | bolus | 402 ± 95.2[a] | –[b] | – | – | – |
| 5.0 | 2 | bolus | 1239[c] (730–1749) | – | – | – | – |
| 7.5 | 4 | bolus | 1290 ± 298 | – | – | – | – |
| 11.5 | 3 | bolus | 2239 ± 114 | 8437 ± 2771 | 7.05 ± 2.49 | 23.6 ± 6.4 | 23.0 ± 8.1 |
| 19 | 3 | bolus | 2928 ± 502 | 8198 ± 2783 | 7.37 ± 0.83 | 40.8 ± 11.6 | 17.9 ± 1.5 |
| 32 | 3 | 30 min | 5332 ± 936 | 46 318 ± 16 408 | 20.4 ± 6.38 | 12.4 ± 4.1 | 17.4 ± 2.1 |
| 50 | 5 | 30 min | 6727 ± 2096 | 32 792 ± 10 075 | 13.5 ± 3.06 | 27.7 ± 9.7 | 25.7 ± 8.0 |
| 65 | 1 | 30 min | 6126 | 31 782 | 11.5 | 34.0 | 26.4 |
| 75 | 3 | 30 min | 8173 ± 126 | 63 911 ± 21 853 | 19.6 ± 15.3 | 21.7 ± 7.1 | 27.7 ± 19.3 |

[a]Values are mean ± s.d. [b]Insufficient data to calculate parameter. [c]Values are mean (range).

**Table 4** Haematological toxicity of BMS-181174: worst WHO grade toxicity

| Dose | No. of patients | Haemoglobin | | | | | Neutrophils | | | | | Platelets | | | | |
|---|---|---|---|---|---|---|---|---|---|---|---|---|---|---|---|---|
| | | 0 | 1 | 2 | 3 | 4 | 0 | 1 | 2 | 3 | 4 | 0 | 1 | 2 | 3 | 4 |
| 0.8 | 4 | 2 | 1 | | 1 | | 3 | | 1 | | | 4 | | | | |
| 1.6 | 3 | 2 | 1 | | | | 3 | | | | | 3 | | | | |
| 3.2 | 7 | 2 | 2 | 2 | 1 | | 7 | | | | | 7 | | | | |
| 5.0 | 5 | 2 | 1 | 2 | | | 5 | | | | | 5 | | | | |
| 7.5 | 5 | 3 | 2 | | | | 5 | | | | | 5 | | | | |
| 11.5 | 7 | 3 | 3 | 1 | | | 7 | | | | | 7 | | | | |
| 19 | 8[a] | 1 | 5 | 1 | | | 7 | | | | | 7 | | | | |
| 32 | 5 | 4 | 1 | | | | 4 | 1 | | | | 4 | | 1 | | |
| 50 | 6 | 3 | | 3 | | | 6 | | | | | 6 | | | | |
| 65 | 12 | 1 | 4 | 6 | | 1 | 10 | | 2 | | | 6 | 2 | 2 | | 2 |
| 75-U | 14 | 3 | 3 | 7 | 1 | 0 | 10 | 1 | 2 | 0 | 1 | 7 | 1 | 3 | 0 | 3 |
| 75-T | 6 | 0 | 2 | 4 | 0 | 0 | 1 | 1 | 2 | 2 | 0 | 1 | 0 | 0 | 2 | 3 |

75-U, previously untreated patients at dose level 75 mg m⁻²; 75-T, patients who had received prior chemotherapy followed by BMS-181174 at dose level 75 mg m⁻². [a]One patient no data.

**Figure 2** Pharmacokinetics of total N7-[2-thioethyl]-MMC (TEMMC), derived from BMS-181174 in vivo. (**A**) Representative plasma concentration–time profile of TEMMC from a patient receiving BMS-181174 at 65 mg m⁻² via a 30-min intravenous infusion. (**B**) Assessment of dose linearity of TEMMC after administration of BMS-181174

© Cancer Research Campaign 1998

their nominal value, the within day error was ≤9% relative standard deviation (RSD), and the between day error was ≤3% RSD. For the modified analytical method, the predicted QC concentrations for TEMMC were within ±9% of their nominal values, with the exception of the 214 ng ml$^{-1}$ QC, which deviated by –20%. This QC was made at the time of the twelfth analytical session and, because of the large deviation, was replaced with a QC of 100 ng ml$^{-1}$. The overall within day error was ≤9% RSD, and the between day error was ≤11% RSD. The QC data indicated adequate accuracy and precision during the analysis.

Several of the study samples for one patient at the 75 mg m$^{-2}$ dose level appeared to be haemolysed. The plasma concentration–time profile fluctuated wildly, so a pharmacological analysis was not performed. Thus, at the 75 mg m$^{-2}$ dose level, the mean pharmacokinetic parameters of the remaining three patients are reported. The results are shown in Table 3. The assay was not sufficiently sensitive to quantify plasma levels generated by treatment at the 0.8 and 1.6 mg m$^{-2}$ dose levels. At dose levels of 3.2–7.5 mg m$^{-2}$, there was insufficient data for pharmacokinetic analysis, therefore only $C_{MAX}$ is reported (Table 3). Pharmacokinetic data were obtained at doses of 11.5–75 mg m$^{-2}$. At 11.5 and 19 mg m$^{-2}$, the estimated $T_{1/2}$ was approximately 7 h. Using the 30-min infusional schedule, the disappearance of TEMMC from plasma appeared to be biphasic, with an initial rapid distribution phase followed by a mean $T_{1/2}$ ranging from 11.5 to 20.4 h. A representative plasma concentration–time curve of TEMMC is shown in Figure 2A. The AUC(INF) increased in a linear fashion with dose over the range 11.5–75 mg m$^{-2}$ (Figure 2B). $T_{1/2}$ apparent CLT and apparent $V_{SS}$ did not change in relation to dose (Table 3). These findings suggest that the pharmacokinetics of TEMMC are linear after the administration of BMS-181174 doses ranging from 11.5 to 75 mg m$^{-2}$. Mean apparent CLT values ranged from 12.4 to 40.8 ml min$^{-1}$ m$^{-2}$, and mean apparent $V_{SS}$ values ranged from 17.4 to 27.71 m$^{-2}$. The apparent $V_{SS}$ of TEMMC is equivalent to total body water, suggesting extensive distribution of TEMMC.

**Figure 3** Effect of BMY25067 on granulocyte and platelet counts. This case shows worsening thrombocytopenia after successive courses of BMS-181174 in a previously treated patient (case 62) with metastatic colorectal cancer

**Table 5** Thrombophlebitis caused by BMS-181174

| Dose level (mg m$^{-2}$) | No. of patients | Thrombophlebitis, worst grade | | | | |
|---|---|---|---|---|---|---|
| | | 0 | 1 | 2 | 3 | 4 |
| 0.8 | 4 | 4 | | | | |
| 1.6 | 3 | 3 | | | | |
| 3.2 | 7 | 7 | | | | |
| 5.0 | 5 | 5 | | | | |
| 7.5 | 5 | 3 | 1 | 1 | | |
| 11.5 | 7 | 5 | | 1 | 1 | |
| 19.0 | 8 | 7 | 1 | | | |
| 32.0 | 5 | 2 | 1 | 2 | | |
| 50.0 | 6 | 6 | | | | |
| 65.0 | 12 | 7 | 0 | 4 | 1 | 1 |
| 75.0 | 20 | 12 | 0 | 3 | 3 | 2 |

### Toxicity

### *Haematological toxicity*

Table 4 describes the haematological toxicity of BMS-181174. Apart from the development of grade 2–3 anaemia in several patients treated at 3.2–5 mg m$^{-2}$, which may have been at least in part disease related, there was no significant (grade ≥3) haematological toxicity up to 50 mg m$^{-2}$ (see Table 4). Twelve patients, all pretreated with chemotherapy, received BMS-181174 at dose level 65 mg m$^{-2}$. No grade 3 toxicity was seen at this dose level, but grade 4 anaemia was seen in one case (8%) and thrombocytopenia in two cases (17%), both of whom received prophylactic platelet transfusion (see Figure 3 for illustration of one of these patients, case 62). No patients at this dose level required broad-spectrum intravenous antibiotics for neutropenic sepsis. Two patients had intravenous cefuroxime in the absence of evidence of neutropenia, one for a chest infection and the second at his local hospital for a severe episode of pneumonia/pneumonitis from which he died (case 72, see below).

Of 20 patients treated at the highest dose level (75 mg m$^{-2}$), 14 had no prior chemotherapy. In this group, grade 3–4 anaemia, neutropenia and thrombocytopenia were experienced by one (7%), one (7%) and three (21%) patients respectively. Equivalent figures for the six previously treated patients at this dose level were none, two (33%) and five (83%) respectively. Thus, at this dose level, a total of eight patients had grade 4 thrombocytopenia, which necessitated prophylactic platelet transfusion in four, treatment delay in one case, dose reduction in another and cessation of treatment in five cases. Similar to the previous dose level, neutropenia did not present a problem and no patients required antibiotics for neutropenic sepsis.

Nadir blood counts were experienced after a median of 15–22 days. At the highest dose level there did not appear to be a clinically significant difference in time to nadir counts between previously treated and untreated patients (not shown). After treatment at 65 mg m$^{-2}$, granulocyte counts recovered to normal in 34.5 days (median) (range 27–42 days) and platelet counts in 25 days (range 21–45 days). Equivalent figures for recovery after treatment at 75 mg m$^{-2}$ were 22.5 days (range 19–43) and 29.5 days (range 20–46), with no clinically significant difference between treated and untreated patients. In patients receiving multiple courses, there was a tendency for each successive course to be followed by a more profound nadir, with a longer time to recovery (see Figure 3).

### *Thrombophlebitis*

Of 31 patients treated at 7.5–50 mg m$^{-2}$, eight (26%) had thrombophlebitis, although this was generally mild (grade 1–2; see Table 5). However 7 out of 32 (22%) evaluable patients treated at

 *© Cancer Research Campaign 1998*

**Table 6** Cardiotoxicity: effect of BMS-181174 on LVEF

| Dose | No. of patients | NE | NC | Reduction |
|---|---|---|---|---|
| 0.8 | 4 | 3 | 1 | 0 |
| 1.6 | 3 | 1 | 1 | 1 |
| 3.2 | 7 | 0 | 5 | 2 |
| 5.0 | 5 | 1 | 3 | 1 |
| 7.5 | 5 | 0 | 5 | 0 |
| 11.5 | 7 | 2 | 5 | 0 |
| 19 | 8 | 3 | 5 | 0 |
| 32 | 5 | 1 | 3 | 1 |
| 50 | 6 | 2 | 3 | 1 |
| 65 | 12 | 3 | 9 | 0 |
| 75 | 20 | 4 | 13 | 3 |

Significant reduction in LVEF defined as decline from baseline LVEF by ≥10% taking the value to below normal (50%), or a fall of ≥5% below normal (i.e. ≤45%). NE, not evaluable; NC, no change.

**Table 7** Responses to BMS-181174

| Dose level (mg $m^{-2}$) | No. of patients | Response | | | | |
|---|---|---|---|---|---|---|
| | | NE | PD | SD | MR | PR |
| 0.8 | 4 | | 4 | | | |
| 1.6 | 3 | | 3 | | | |
| 3.2 | 7 | 1 | 5 | 1 | | |
| 5.0 | 5 | 1 | 4 | | | |
| 7.5 | 5 | | 4 | | | 1 |
| 11.5 | 7 | | 5 | 2 | | |
| 19.0 | 8 | | 7 | 1 | | |
| 32.0 | 5 | | 5 | | | |
| 50.0 | 6 | | 5 | 1 | | |
| 65.0 | 12 | 2 | 8 | | 1 | 1 |
| 75.0 | 20 | 4 | 10 | 4 | | 2 |
| Total | 82 | 10 | 59 | 8 | 1 | 4 |

NE, not evaluable; PD, progressive disease; SD, stable disease; MR, minor response; PR, partial response.

65–75 mg $m^{-2}$ experienced thrombophlebitis of grade 3–4. Three of these had grade 4 thrombophlebitis, which presented in two cases as axillary vein thrombosis leading to cessation of therapy. The third patient (case 72, treated at 65 mg $m^{-2}$) developed peripheral grade 3 thrombophlebitis after one course, and the second and third were administered through a long line inserted via the contralateral antecubital fossa. Acute dyspnoea developed on day 15, with ventilation/perfusion scan evidence of pulmonary embolus (i.e. grade 4 toxicity). Despite anticoagulation and a normal peripheral white blood count, he deteriorated with a radiological picture of fulminating pneumonitis (not confirmed microbiologically), and he died on day 27 of course three. Permission for post-mortem examination was declined by his family (see below, Pulmonary toxicity).

### *Cardiotoxicity*

Table 6 shows the effects of BMS-181174 on LVEF. Twenty patients who received only one course of BMS-181174 were not evaluable because they did not undergo repeat LVEF assessment. In one patient treated at 1.6 mg $m^{-2}$, the LVEF fell from 63% pretreatment to 35% after three courses, recovering to 53% 3 months after the sixth course. This patient had previously undergone radiotherapy to a paratracheal mass; subsequently, we excluded patients who had received chest radiotherapy involving the cardiac field.

Significant reduction in LVEF was seen in 9 out of 62 (15%) evaluable patients, including individual patients treated at most dose levels between 1.6 and 65 mg $m^{-2}$ and three of sixteen evaluable patients (19%) treated at 75 mg $m^{-2}$ (Table 6). Overall, there was no indication of a relationship with dose or cumulative dose for this toxicity. The nadir LVEF value was observed after a median of two courses (range one to six courses). Of the nine patients experiencing a drop in LVEF, four had repeat echocardiography, which showed recovery of left ventricular function in 1 week to 3 months. In one patient, a second course of treatment was delayed for 2 weeks until the LVEF had recovered, and, in the remaining three, treatment was discontinued for other reasons.

**Table 8** Reasons for stopping treatment

| Dose level (mg $m^{-2}$) | No. of patients | Reasons for stopping treatment | | | | | |
|---|---|---|---|---|---|---|---|
| | | PD[a] | Toxicity | | | | Completed six courses |
| | | | Haematological | Lung | Thrombophlebitis/ thrombosis | Other | |
| 0.8 | 4 | 4 | | | | | |
| 1.6 | 3 | 2 | | | | | 1 |
| 3.2 | 7 | 7 | | | | | |
| 5.0 | 5 | 5 | | | | | |
| 7.5 | 5 | 5 | | | | | |
| 11.5 | 7 | 5 | | | | | 2 |
| 19.0 | 8 | 7 | | | | | 1 |
| 32.0 | 5 | 4 | 1 | | | 1 | |
| 50.0[a] | 6 | 5 | | | | 2 | |
| 65.0[a] | 12 | 10 | 1 | 1 | 1 | 1 | |
| 75.0[a] | 20 | 12 | 6 | 3 | 2 | 2 | |

[a]Includes death on treatment.

© Cancer Research Campaign 1998 

The change in LVEF was asymptomatic in all patients, with the exception of one who developed grade 3 dyspnoea 3 months after a single course of BMS-181174 at 75 mg $m^{-2}$. This patient had a normal chest radiograph and an echocardiogram that showed septal hypertrophy with an LVEF of 45% compared with 66% pretreatment. The patient discontinued treatment because of thrombocytopenia (see below) and the echocardiogram was not repeated.

### *Pulmonary toxicity*

As described above, one patient (case 72) died having developed pulmonary embolism followed by a clinical picture of pneumonitis. Two further patients (cases 55 and 57) died suddenly having developed pneumonia without documented neutropenia within 1 month of receiving BMS-181174, in both cases after three courses at 75 mg $m^{-2}$. In one of these cases, post-mortem analysis showed adenocarcinoma of the lung with focal early adult respiratory distress syndrome (ARDS).

Among the patients whose LVEF did not change significantly by the criteria used here, there was one further case of possible drug-related dyspnoea. A patient who received two courses of BMS-181174 at 75 mg $m^{-2}$ developed grade 3 dyspnoea with a normal chest radiograph. The echocardiogram showed a small pericardial effusion, with reduction in LVEF to 58% from a baseline value of 73%.

### *Nephrotoxicity*

Possible renal toxicity was noted in three of 82 patients (4%). This was manifest as a rise in serum creatinine from the baseline value, amounting to grade 1 toxicity in one patient treated at 65 mg $m^{-2}$ and two cases of grade 2 toxicity, one at 32 mg $m^{-2}$ and one at 50 mg $m^{-2}$. The latter (case 47) was a patient with metastatic transitional cell carcinoma of the bladder and unilateral hydronephrosis. He was treated with two cycles of BMS-181174 at 50 mg $m^{-2}$, having previously received combination chemotherapy including cisplatin. His serum creatinine before BMS-181174 was borderline at 123 μmol $l^{-1}$, rising after a second course to a peak of 350 μmol $l^{-1}$. He had trace haematuria and proteinuria, and chemotherapy was discontinued. He developed clinical evidence of progressive disease and died 7 weeks after the second course of BMS-181174. No antemortem or post-mortem renal histology was available.

### *Symptomatic toxicities*

BMS-181174 caused mild/moderate emesis that was generally well-controlled with standard antiemetics. Grade 1–2 nausea was reported by 21 patients including a few patients at all dose levels ≥3.2 mg $m^{-2}$. Moderate or severe emesis (grade 2–3) was experienced by fifteen patients including thirteen treated at 65–75 mg $m^{-2}$. One patient experienced a mild allergic reaction with facial flushing, dizziness and throat dryness/tightness during the first injection of BMS-181174. The treatment was stopped and the patient recovered over 1 hour without additional medication.

### Response to BMS-181174

Four patients achieved a partial response (PR, reduction in size of marker lesion(s) to <50% of pretreatment measurement) giving a response rate of 4 out of 82 (5%) (Table 7). Of 50 patients treated at 0.8–50 mg $m^{-2}$ BMS-181174, only one (2%) responded, achieving a PR in liver metastases from colorectal cancer after four courses at 7.5 mg $m^{-2}$. Of 12 patients treated at 65 mg $m^{-2}$, there was one minor response (MR, reduction of 25–50% from baseline) in pulmonary metastases from colorectal cancer after two courses and one PR in a patient with ovarian cancer. Both patients had previously received systemic therapy, with 5-fluorouracil/folinic acid and platinum-containing chemotherapy respectively. Of 20 patients treated at 75 mg $m^{-2}$, one patient with adenocarcinoma of unknown primary site achieved PR in pulmonary and liver metastases after two courses and one patient with non-small-cell lung cancer achieved PR in lung deposits, again after two courses. Neither of these patients had prior chemotherapy. Thus the PR rate at 65–75 mg $m^{-2}$ was 3 out of 32 (9%). Although this was higher than the rate seen at low dose levels (2% at 0.8–50 mg $m^{-2}$), there was no significant difference between response rates in these two treatment groups ($P = 0.29$ by Fisher's exact test).

### Reasons for stopping treatment, treatment-related deaths

Of the 82 patients treated, only four (5%) were able to complete six courses of treatment, at 1.6 mg $m^{-2}$ (one case), 11.5 mg $m^{-2}$ (two cases) and 19 mg $m^{-2}$ (one case). Treatment was discontinued before completion of six courses because of progressive disease in 44 of 50 patients (88%) treated at 0.8–50 mg $m^{-2}$ (see Table 8). At 65–75 mg $m^{-2}$, treatment was discontinued for progressive disease in 22 out of 32 patients (69%) and because of toxicity in 17 out of 32 (53%). In seven cases, both toxicity and disease progression were factors in the decision to stop treatment. Non-haematological toxicities leading to cessation of treatment included grade 3 thrombophlebitis (one case), deep venous thrombosis (i.e. grade 4 thrombophlebitis, two cases), pulmonary toxicity, including pneumonitis/pneumonia (three cases), pulmonary embolus (one case), unexplained dyspnoea (one case), cardiac toxicity (one case), renal impairment (one), anaphylaxis (one) and declining performance status (one).

As described above, there were four possible treatment-related deaths. Three of these showed evidence of pulmonary toxicity. Two patients (cases 55 and 57) died suddenly with a clinical picture of pneumonia/pneumonitis, in both cases after three courses at 65 mg $m^{-2}$. In a third (case 72), a similar clinical picture developed after pulmonary embolism possibly related to thrombosis around a long line. Finally, one patient with bladder cancer (case 47) died 7 weeks after a second course of BMS-181174 at 50 mg $m^{-2}$. There was evidence of progressive disease, but deteriorating renal failure may have been a contributory factor.

### DISCUSSION

For clinical testing, the starting dose was chosen at 0.8 mg $m^{-2}$, which corresponds to one-third of the TLD in the dog. Preclinical studies had noted that the dog was more sensitive to this drug than mice or rats (Nicaise and Usakewicz, 1989). The lack of clinical activity or haematological toxicity observed in patients treated at the early dose levels (0.8–7.5 mg $m^{-2}$) suggested that this dose was inappropriately low. Pharmacokinetic analysis seemed to confirm this impression: despite development of a sensitive assay it was not possible to detect accurately quantifiable levels of the TEMMC moiety of BMS-181174 in the plasma of patients treated at the 0.8 and 1.6 mg $m^{-2}$ dose levels. No pharmacokinetic information had been collected in the single-dose toxicity study in dogs. However, in mice, at the $LD_{10}$ dose, the pharmacokinetics of TEMMC were determined and the AUC was 47 646 ng h $ml^{-1}$ (LaCreta, 1995*b*). Therefore, the AUC at the $LD_{10}$ in mice was

© Cancer Research Campaign 1998

similar to the AUC at doses ranging from 32 to 75 mg $m^{-2}$ in humans. This finding supports the belief that the early doses were inappropriately low.

The data obtained on patients treated with bolus injections at 11.5–19 mg $m^{-2}$ showed an estimated $T_{1/2}$ of approximately 7 h. From 32 mg $m^{-2}$ the mode of administration was changed from bolus injection to short (30 min) infusion in an attempt to reduce the risk of thrombophlebitis. Sample analysis indicated that the pharmacokinetics were linear at doses of 11.5–75 mg $m^{-2}$, and the drug appeared to be widely distributed.

The dose-limiting toxicity of BMS-181174 was myelosuppression. Significant (grade 3–4) myelosuppression necessitating treatment modification was seen at 65 and 75 mg $m^{-2}$. In general, neutropenia was less of a problem than thrombocytopenia, which was frequently prolonged and cumulative. This was the commonest toxicity necessitating cessation of treatment in patients receiving multiple courses of BMS-181174 at 65–75 mg $m^{-2}$. Median nadir times for neutropenia were 15–19 days, while nadirs for thrombocytopenia were later (16–24 days), as observed with the parent drug MMC (Crooke and Bradner, 1976). At 75 mg $m^{-2}$ there was a notably higher incidence of grade 3–4 thrombocytopenia in patients previously treated with chemotherapy (83%) compared with previously untreated patients (21%). There was also a difference in incidence of grade 3–4 neutropenia: 33% in pretreated cases compared with 7% untreated. This suggests that the MTD for chemotherapy-naive patients is 75 mg $m^{-2}$ and for pretreated patients 65 mg $m^{-2}$.

Thrombophlebitis of grades 1–3 was seen in 13% of patients treated with BMS-181174 at dose levels 0.8–19 mg $m^{-2}$. In an attempt to ameliorate this, the schedule of administration was altered from bolus injection to 30-min infusion. Paradoxically, this may have been at least partly responsible for the more severe thrombophlebitis seen at higher dose levels. Study of the effects of similar doses of BMS-181174 administered by 6-h infusion have shown local venous toxicity with thrombophlebitis and venospasm during the infusion (Planting et al, 1994). None of our patients were treated via a central line; while this might protect against peripheral thrombophlebitis, it could increase the risk of central thrombosis/embolism. Indeed, this contributed to the death of one of our patients who was treated via a long line. Other side-effects of BMS-181174 included nausea and vomiting, which was generally mild and well controlled with standard antiemetics, usually dexamethasone and metoclopramide.

We observed four cases of serious organ toxicity. There were three cases of possible drug-induced pneumonitis associated in each case with rapid clinical deterioration and death. This toxicity would represent a serious obstacle to further clinical testing of BMS-181174. There was also evidence of an effect on renal function. The parent drug MMC is a known cause of pulmonary fibrosis and reportedly causes a rise in serum creatinine in 2% of patients. Haemolytic–uraemic syndrome can occur and is particularly likely in patients who receive a cumulative dose of over 60 mg (Martino et al, 1979; Ratanatharathorn et al, 1979; Rabadi et al, 1982). Preclinical studies have shown that BMS-181174 is cardiotoxic in rats, although not in mice or dogs (Dorr et al, 1992). In our phase I study we saw some evidence of impairment of cardiac function as measured by changes in LVEF. In one patient, previous radiotherapy involving the cardiac field may have contributed to an increased susceptibility to BMY-related cardiotoxicity. Otherwise the changes in LVEF were generally mild, asymptomatic, reversible and with no clear dose relationship.

We were able to demonstrate that BMS-181174 has anti-tumour activity. Responses were documented amongst previously treated and untreated patients. The low overall response rate (6%) reflects the fact that the starting dose was, in retrospect, at least an order of magnitude too low. At the highest dose levels (65–75 mg $m^{-2}$) BMS-181174 achieved responses in cases of colorectal cancer, ovarian cancer, non-small-cell lung cancer and an adenocarcinoma of unknown primary. Thus, the spectrum of activity was similar to that of MMC (Carter and Crooke, 1979). However, the toxicity profile appeared to be no more favourable than that of the parent drug, and no phase II studies are planned.

### REFERENCES

Bradner WT, Rose WC, Schurig JE and Florczyk AP (1990) Antitumour activity and toxicity in animals of N-7[2-(4-nitrophenyldithio)ethyl] mitomycin C (BMY-25067). *Invest New Drugs* **8**: S1–S7

Bregman CL, Buroker RA, Bradner WT, Hirth RS and Madisoo H (1989) Cardiac, renal and pulmonary toxicity of several mitomycin derivatives in rats. *Fundament Appl Toxicol* **13**: 46–64

Carter SK and Crooke ST (eds) (1979) *Mitomycin C, Current Status and New Developments.* Academic Press: New York

Crooke ST and Bradner WT (1976) Mitomycin C: a review. *Cancer Treat Rev* **3**: 121–139

Dorr RT, Shipp NG, Liddil JD, Iyengar BS, Kunz KR and Remers WA (1992) Cardiotoxicity of mitomycin A, mitomycin C and seven $N^7$ analogs in vitro. *Cancer Chemother Pharmacol* **31**: 1–5

Doyle TW and Vyas DM (1990) Second generation analoges of etoposide and mitomycin C. *Cancer Treat Rev* **17**: 127–131

Feigenbaum H (1994) *Echocardiography*, 5th edn. Lea and Febiger: Philadelphia

Gaver RC (1995) *A High-performance Liquid Chromatographic Procedure for the Quantitation of N7-[2-Thioethyl]-Mitomycin C Derived from BMS-181174 (BMY-25067) in Human Plasma.* Bristol-Myers Squibb Pharmaceutical Research Institute, Accession no. 910049139

Gibaldi M and Perrier D (1982) Noncompartmental analysis based on statistical moment theory. In *Pharmacokinetics*, 2nd edn, pp. 409–416. Marcel Dekker: New York

La Creta FP (1995*a*) *A High Performance Liquid Chromatographic Procedure for the Quantitation of N7-[2-Thioethyl]-Mitomycin C, Derived from BMS-181174, in Human and Mouse Plasma.* Bristol-Myers Squibb Pharmaceutical Research Institute, Accession no. 910049167

LaCreta FP (1995*b*) *Exploratory Pharmacokinetic Study of BMS-181174 (BMY-25067) in Mice.* Bristol-Myers Squibb Pharmaceutical Research Institute Report no. 50950

Martino S, Baker LH, Pollard RJ, Correa JJ and DeMattia MD (1979) Pulmonary toxicity of Mitomycin C. In *Mitomycin C, Current Status and New Development*, Carter SK and Crooke ST. (eds), pp. 231–242. Academic Press: New York

Nicaise C and Usakewicz J (1989) *BMY-25067, Basic Data Brochure.* Bristol-Myers Squibb Company Pharmaceutical Research and Development Division, Wallingford, CT, USA

Planting A, van der Berg M, van der Gaast A, Stoter G, de Boer-Dennert M, Dewji R, Santabarbara P, Kolker H, Schellens J and Verweij J (1994) Phase I study of BMY-25067 as a 6-hour infusion every 4 weeks in patients (pts) with solid tumors. In *Proceedings of the 8th NCI-EORTC Symposium on New Drugs in Cancer Therapy, 15–18 March, 1994,* Abstract 258. *Ann Oncol* **5** (suppl. 5)

Rabadi SJ, Khandekar JD and Miller JH (1982) Mitomycin-induced haemolytic uraemic syndrome: case presentation and review of the literature. *Cancer Treat Rep* **66**: 1244–1247

Ratanatharathorn V, Baker LH, Cadnapaphornchai P, Rosenberg BF and Vaitkevicius VK (1979) Clinical and pathological study of Mitomycin C nephrotoxicity. In: *Mitomycin C, Current Status and New Development*, Carter SK and Crooke ST. (eds), pp. 219–229. Academic Press: New York

Rockwell S, Kemple B and Kelley M (1995) Cytotoxicity of BMS-181174. Effects of hypoxia, dicoumarol, and repair deficits. *Biochem Pharmacol* **50**: 1239–1243

Singh SV, Xu BH, Gupta V, Emerson EO, Zaren HA and Jani JP (1995) Characterisation of a human bladder cancer cell line selected for resistance to BMS-181174, a novel analogue of mitomycin C. *Cancer Lett* **95**: 49–56

Teichholtz LE, Kreulen T, Herman MV and Gorlin R (1976) Problems in echocardiographic volume determinations: echocardiographic-angiographic correlations in the presence or absence of asynergy. *Am J Cardiol* **37**: 7–11

WHO (1979) *WHO Handbook for Reporting Results of Cancer Treatment.* WHO Offset Publication no. 48. World Health Organization: Geneva

© Cancer Research Campaign 1998